# The importance of close follow-up in the postpartum period. Manic episode after childbirth in a patient with bipolar disorder. A case report

**DOI:** 10.1192/j.eurpsy.2025.2401

**Published:** 2025-08-26

**Authors:** P. Del Sol, A. Izquierdo, R. Fernández, M. García Moreno, B. Tejero, M. Diaz de Neira

**Affiliations:** 1 Hospital Puerta de Hierro, Madrid; 2 Hospital Infanta Cristina, Parla, Spain

## Abstract

**Introduction:**

Bipolar disorder is a serious mental disorder that requires follow-up and pharmacological treatment for the patient to function properly. It is known that stressful events can generate decompensation in these patients. Pregnancy and postpartum are moments of high vulnerability for women with bipolar disorder, being essential their immediate follow-up and treatment in case of decompensation. In these cases it is essential to ensure an adequate bond with the baby.

**Objectives:**

To present a case of a patient with a diagnosis of bipolar disorder, who in both pregnancies has a manic episode after delivery.

**Methods:**

Case presentation and literature review.

**Results:**

The patient is a 37-year-old woman who comes to the emergency department for manic symptoms. Her psychiatric history includes a diagnosis of bipolar disorder in 2022 after an admission for a manic episode two months after the birth of her first child. She is currently under active follow-up by psychiatry and a perinatal group intervention program. She lives with her husband and two-year-old son.

During pregnancy she received lithium without decompensation. When she came to the emergency room, she was on lithium 400 mg every 8 hours and olanzapine 10 mg prescribed 4 weeks ago by her psychiatrist, who had noticed the decompensation, which she stopped a week ago on her own.

In the examination, he shows an accelerated speech with verbose and uninhibited contact. She says that 5 days ago she began to present the idea that she was a threat to her children, with increasing anguish. She began with a decrease in the hours of sleep and suddenly refers that the idea of having to travel to Bali appeared, since “it is her favorite place in the world”. Without considering the price and without informing her husband, she left her home and arrived in Bali, where she incurred high expenses. When she arrived there, she reported meeting a friend who helped her to return to her country and informed her family.

**Conclusions:**

Pregnancy and postpartum are moments of high vulnerability and emotional intensity for any woman. It is known that for patients with mental disorders, this vital moment may involve a risk of decompensation of their mental disorder, compromising their health, and may have negative repercussions on the bonding with their baby and the style of attachment they build with it. It is essential to train professionals in pharmacological treatments allowed during pregnancy, postpartum and lactation, as well as to facilitate close follow-up programs for these patients in order to reduce the risk of decompensation as much as possible.

**Disclosure of Interest:**

None Declared

